# Micronutrient intake and the probability of nutrient adequacy among children 9–24 months of age: results from the MAL-ED birth cohort study

**DOI:** 10.1017/S1368980020000877

**Published:** 2021-06

**Authors:** DA Antiporta, R Ambikapathi, A Bose, B Maciel, TC Mahopo, C Patil, A Turab, MP Olortegui, M Islam, A Bauck, BJJ McCormick, LE Caulfield, MAL-ED Network Investigators

**Affiliations:** 1 Department of Epidemiology, The Johns Hopkins Bloomberg School of Public Health, Baltimore, MD, USA; 2 Fogarty International Center, National Institutes of Health, Bethesda, MD, USA; 3 Department of Community Health, Christian Medical College, Vellore, Tamil Nadu, India; 4 Department of Nutrition, Universidade Federal do Rio Grande do Norte, Natal, Rio Negro, Brazil; 5 Department of Nutrition, University of Venda, Thohoyandou, South Africa; 6 Department of Women, Children and Family Health Sciences, University of Illinois-Chicago, Chicago, IL, USA; 7 Centre of Excellence in Women and Child Health, Aga Khan University, Karachi, Pakistan; 8 AB PRISMA, Iquitos, Loreto, Peru; 9 icddr,b, Dhaka, Bangladesh; 10 Department of International Health, The Johns Hopkins Bloomberg School of Public Health, Baltimore, MD 21205, USA

**Keywords:** Child nutrition, Diet, Micronutrients, Cohort studies

## Abstract

**Objective:**

To estimate the total energy and micronutrient intakes of children 9–24 months of age and evaluate the probability of adequacy (PA) of the diet in seven MAL-ED sites.

**Design:**

Cohort study. Food intake was registered monthly using 24-h recalls beginning at 9 months. We estimated PA for thirteen nutrients and overall mean PA (MPA) by site and 3-month periods considering estimated breast milk intake.

**Setting:**

Seven sites in Asia, Africa and Latin America.

**Participants:**

1669 children followed from birth to 24 months of age.

**Results:**

Median estimated %energy from breast milk ranged from 4 to 70 % at 9–12 months, and declined to 0–39 % at 21–24 months. Iron bioavailability was low for all sites, but many diets were of moderate bioavailability for zinc. PA was optimal for most nutrients in Brazil and South Africa, except for iron and vitamin E (both), calcium and zinc (South Africa). PA for zinc increased only for children consuming a diet with moderate bioavailability. MPA increased 12–24 months as the quantity of complementary foods increased; however, PA for vitamin A remained low in Bangladesh and Tanzania. PA for vitamins D and E and iron was low for most sites and age groups.

**Conclusions:**

MPA increased from 12 to 24 months as children consumed higher quantities of food, while nutrient density remained constant for most nutrients. Ways to increase the consumption of foods containing vitamins D, E and A, and calcium are needed, as are ways to increase the bioavailability of iron and zinc.

Infants and young children require a diet with adequate energy and nutrient intakes to support growth and development^(1)^. It is recommended that breast milk should be the sole source of nutrition for the infant from birth to 6 months of age, after which nutrient-rich foods can be fed along with breast milk to meet the growth and developmental needs of the child^(2)^. However, breast milk’s contribution to the diet diminishes over time, as the child moves from their first solid foods to the family diet; eventually, breast milk is no longer part of the child’s diet^(1)^.

In low-resource settings, growth faltering typically begins to emerge during complementary feeding. Research and programmatic efforts have worked to improve the quantity and quality of non-breast milk foods fed to children to alter the pathway leading to stunting^(3)^. These efforts are limited by a general lack of studies quantifying the energy and nutrients supplied by complementary foods; this study aimed to fill this gap by providing a fuller characterisation of dietary inadequacies among young children using repeated 24-h recall data over the first and second years of life.

‘The Etiology, Risk Factors, and Interactions of Enteric Infections and Malnutrition and the Consequences for Child Health and Development Project’ (MAL-ED) was a multisite birth cohort study investigating the role of enteropathogen exposure, poor diet and environmental enteric dysfunction as they may impact child growth and development over the first 24 months of life^(4)^. It took place in four sites in South Asia (Dhaka, Bangladesh (BGD); Vellore, India (INV); Bhaktapur, Nepal (NEB); Naushero Feroze, Pakistan (PKN)), two sites in Latin America (Fortaleza, Brazil (BRF); Loreto, Peru (PEL)) and two sites in Sub-Saharan Africa (Venda, South Africa (SAV); Haydom, Tanzania (TZH)). Beginning at 9 months and continuing monthly through 24 months, caregivers were queried about the intake of non-breast milk foods fed and consumed by their child on the previous day using a harmonised 24-h recall methodology^(5)^. The objectives of this study were to quantify the micronutrient intakes of these children, estimate the nutrients provided by breast milk (among those breastfed), and evaluate the overall probability of micronutrient adequacy of the diet over time. A similar approach taken here has already been applied to the data from the Nepal site^(6)^; therefore, we report findings from the remaining seven sites.

## Methods

The sites for the MAL-ED study were chosen based on the infectious disease and malnutrition rates and feasibility considerations, rather than infant feeding practices or dietary patterns^(4)^. Each site enrolled and followed a cohort of over 200 children from near birth until 24 months of age, leading to an overall enrolment of 1905 children. Enrolment was staggered monthly to reduce seasonal patterns, and the study was implemented from November 2009 until February 2014. Enrolled infants were ≤17 d old, born singleton with a birth weight >1500 g, without serious illnesses, to a mother at least 16 years of age, and to a family intending to stay in the community for at least 6 months^(4)^. All sites received ethical approval, as appropriate, from governmental, local and collaborating institutional review boards. Signed informed consent was obtained from the parent/guardian of each participating child.

### Infant feeding

The overall methodology for data collection on infant feeding has been published^(5)^. Beginning at enrolment, caregivers were queried about the initiation of breastfeeding and infant feeding since birth. Thereafter, households were visited twice weekly, and caregivers were asked about breastfeeding and the introduction of non-breast milk liquids (water, juice, teas, other milks) as well as semisolid or solid foods^(5)^. From this information, we calculated the percentage of days a child is breastfed over any time period from birth to 24 months.

Beginning at 9 months, the intake of non-breast milk foods was quantified using the 24-h recall methodology; each month, about 10–15 children were randomly selected to have a secondary recall, 2–7 d later. Overall, this protocol resulted in the collection of 16–17 recalls per child from 9 to 24 months. Whether or not the child was breastfed on that day was also recorded. To harmonise the methodology, the study’s Nutrition Technical Subcommittee created procedures for administering the 24-h recall applicable to all sites. The sites utilised the same 24-h recall form, as well as a form to collect detailed information for preparations, both of which resembled forms used at multiple sites in prior studies. Depending on the site, research staff were trained or re-trained in the methodology, and each site developed appropriate tools to aid in quantifying amounts and recipes. The forms were completed by hand, reviewed by the research staff and entered into a computer. The MAL-ED staff at the Johns Hopkins Bloomberg School of Public Health (JHSPH) processed the 24-h recall data, which involved multiple searches to identify errors, processing recipes to obtain nutrient information, the creation of new food codes and the identification of nutrient information for packaged foods, bush foods and/or local fruits and vegetables. Re-trainings of field staff occurred based on error identification, and communication between sites and the team at JHSPH resolved issues or questions identified during data collection.

Following a common set of principles, the recall data were used to create a MAL-ED food composition table for each site, beginning with an appropriate base food composition table. The following base tables were used by site: (1) SAV: Condensed Food Composition Tables for South Africa^(7)^; (2) TZH: Tanzanian Food Composition^(8)^; (3) BRF: Tabela Brasileira de Composição de Alimentos (TBCA)^(9)^; (4) unpublished food composition table of the Instituto de Investigación Nutricional (IIN, Creed de Kanashiro); (5) BGD: unpublished icddr,b food composition table used in prior paediatric studies; (6) World Food Dietary Assessment and the International Mini-list^(10)^. Other food composition tables, including those from the United States^(11)^, Australia, New Zealand^(12)^, United Kingdom^(13)^, as well as manufacturers’ websites and journal articles were consulted to obtain best estimates for each food or preparation, and to obtain comparable nutrient information across the sites. The food composition tables included both raw and cooked values, with cooked values for individual nutrients determined based on the described cooking method and retention factors as provided by the US Department of Agriculture^(14)^.

### Growth

Beginning at enrolment, the weight and length of the children were measured by trained personnel following a standard protocol, using quality control techniques, electronic scales (e.g. SECA, Detecto) and recumbent length-measuring boards (e.g. UNICEF, ShorrBoard). Thereafter, children were weighed and measured on a monthly basis during home visits. To describe child nutritional status, we calculated *z*-scores for weight-for-age (WAZ)^(15)^.

### Estimation of total intake and usual intake

For analyses, children were divided into the following age groups: 9–12, 13–16, 17–20 and 21–24 months. The groups were chosen to reflect differences in feeding during the first and second years of life, to evaluate the progression of dietary adequacy during the second year and to provide 4–5 recalls per child for the estimation of usual nutrient intakes for each child at each time period. The analyses presented include 1669 infants, 88 % of those enrolled, due to the exclusion of Nepal data.

The majority of children in each site were breastfed for 18–24 months; thus, to estimate the adequacy of their total dietary intake, energy and nutrient intake from breast milk needed to be accounted for. Following the methods used for the Nepal site^(6)^, we estimated the contribution of breast milk to the diet by assuming that on any child-day of intake on which the child was breastfed, the amount of breast milk consumed filled the gap for meeting the predicted energy requirement, which considers the age, sex and weight of the child, growth and a moderate physical activity level^(16)^. The estimated energy intake from breast milk and published energy density of breast milk (2·63592 kJ/g (0·63 kcal/g)) were used to calculate the amount of breast milk consumed on that child-day of intake^(1)^. Using published average values for the nutrient content of mature milk, we imputed intakes of fourteen nutrients (thiamine (B_1_), riboflavin (B_2_), niacin (B_3_), pyridoxine (B_6_), folate (B_9_), cobalamin (B_12_), vitamin A, vitamin C, vitamin D, vitamin E, Ca, Mg, Fe and Zn) from breast milk^(1,17,18)^. Total intake on each child-day was the sum of intake from complementary food and breast milk; child-days of intake with no reported breastfeeding were considered total intake (intake from breast milk = 0).

The estimated usual nutrient intake of each child was derived using mixed models by age group and site^(6,19)^. The data were normalised using Box Cox power transformation with sex, weekday and month as covariates. Given that Box Cox transformation can only be applied to strict positive data, we added a constant (1 × 10^−6^) to each intake when zero values existed in the site–age group data. The mixed model included the estimation of random effect per child, the variance of which represents between-individual variance. The variance of residuals represents within-person variance. After fitting the model, we predicted the total intake for each child-day of intake as the sum of the best linear unbiased estimate (BLUE) and the best linear unbiased predictor (BLUP). As suggested by Dodd *et al*.^(19)^ and Tooze *et al.* ^(20)^, the re-transformation of data was adjusted by a bias factor using the formulas published in Dodd *et al.* ^(19)^. Finally, if the data were modified to make them strict positive, then the constant was subtracted from each value. The mean of each child’s total intake was taken as their estimated usual intake (EUI).

### Nutrient densities and bioavailability

Nutrient densities of non-breast milk foods were also calculated per 4·184 kJ (100 kcal) utilising each child’s observed mean nutrient and energy intake at each age period. The phytate-to-zinc and phytate-to-iron molar ratios were estimated for each child-day of intake, and the mean for each child and for each age period were used to characterise the bioavailability of zinc and iron in the diet, respectively^(21)^.

### Probability of adequacy

Each child’s probability of adequacy (PA) was obtained by determining the location of their EUI on the nutrient requirement distribution for the age period^(22,23)^ and determining the AUC for intakes below that value. The estimated average requirement (EAR) and the standard deviation of each requirement distribution were derived from the published recommended nutrient intakes (RNI) and CV^(6,23)^; except for vitamin E among children 9–12 months old for which adequate intake (AI) was set to be the reference requirement (see Supplemental Table 1 for values used for each nutrient). For zinc, we categorised the intakes within each age period as either low (phytate-to-zinc ratio > 15) or moderate (phytate-to-zinc ratio 5–15) bioavailability^(23)^ and then applied EAR and standard deviation for the corresponding bioavailability. For iron, there were two issues: bioavailability and non-normal iron requirement distribution in these age ranges. The phytate-to-iron ratios were >1 %^(24)^, indicating low bioavailability, except for 5 % of children in PEL between 9 and 12 months, and 5–10 % of children in BRF between 9 and 15 months. Therefore, we estimated the recommended intakes based on a 5 % bioavailability for all age periods and all sites. Because of the non-normal requirement distribution, we used the quantiles of iron requirement distributions published by the Institute of Medicine (IOM)^(25)^ for 9–12 months and 1–2 years, and then approximated the PA. We also calculated the mean PA (MPA) for each child across the fourteen micronutrients as a summary measure of nutrient adequacy.

### Sensitivity analysis

To assess the robustness of our results for EUI, PA and MPA, we performed a sensitivity analysis using only dietary data from complementary foods (non-breast milk foods).

## Results

The enrolment WAZ of infants ranged from –1·41 (PKN) to 0·07 (BRF), and 48–50 % were female across the sites. They were born to mothers with mean age ranging from 24 years (INV) to 29 years (TZH), and median parity of two in most sites, but three in PKN and four in TZH. The mean maternal years of schooling was lowest in BGD (5 years) and PKN (2 years) and highest in SAV (11 years). As reported elsewhere, all but four infants from SAV were breastfed from birth, and the median duration of exclusive breastfeeding ranged from 4 d (PKN) to 140 d (BGD)^(26,27)^. Select characteristics of the sample are provided in Table 1.

**Table 1:** Selected maternal and child characteristics of the sample by site (*n* 1669)

Characteristics	BGD (*n* 234)			BRF (*n* 198)			INV (*n* 232)			PEL (*n* 251)			PKN (*n* 262)			SAV (*n* 254)			TZH (*n* 238)		
	Mean		sd	Mean		sd	Mean		sd	Mean		sd	Mean		sd	Mean		sd	Mean		sd
Female																					
*n*		119			95			126			114			134			127			120	
%		50·9			48·0			54·3			45·4			51·1			50·0			50·4	
Weight for age (*z*-score)	–1·11		0·96	0·07		1·04	–0·92		1·04	–0·42		0·90	–1·41		1·04	–0·30		1·00	–0·28		0·93
Length for age (*z*-score)	–1·02		1·00	–0·81		1·16	–1·02		1·05	–0·99		0·98	–1·37		1·16	–0·71		1·01	–1·02		1·13
Maternal age (years)	24·8		5·0	25·1		5·5	23·9		4·2	24·2		6·2	28·1		5·9	26·8		7·2	28·8		6·6
Maternal education (years)	5·6		2·6	9·1		2·8	7·8		3·1	7·8		2·8	6·3		3·6	10·1		2·0	6·1		1·8
Maternal height (cm)	148·8		5·2	155·5		6·7	151·2		5·2	150·1		5·5	153·4		5·7	158·5		6·8	155·9		5·9
Parity	*n*		%	*n*		%	*n*		%	*n*		%	*n*		%	*n*		%	*n*		%
Parity																					
1	94		40·2	63		31·8	78*		33·9	97		38·7	58		22·1	93		36·6	25		10·5
2	81		34·6	67		33·8	77		33·5	50		19·9	40		15·3	54		21·3	37		15·6
>3	59		25·2	68		34·4	75		32·6	104		41·4	164		62·6	107		42·1	176		74·0

BGD, Dhaka, Bangladesh; BRF, Fortaleza, Brazil; INV, Vellore, India; PEL, Loreto, Peru; PKN, Naushero Feroze, Pakistan; SAV, Venda, South Africa; TZH, Haydom, Tanzania.

* Among 230 women with reported data.

At 9–12 months, children were breastfed nearly every day (median 99 % of days) (Table 2). As children aged, and with the exception of BGD, the median per cent of days breastfed declined, and by 21–24 months, the median per cent of days was 0 for PEL, PKN, INV, SAV and TZH. Energy intakes from complementary foods increased with age, and correspondingly the %energy from breast milk declined. At 9–12 months of age, the median %energy from breast milk ranged from a high of 70 % in BGD to a low of 4 % in TZH. At 24 months, the median %energy from breast milk was estimated at about 39 % in BGD but was at or below 0 % for other sites.

**Table 2:** Infant weight, breastfeeding status, energy intake from complementary foods and projected %energy from breast milk by age and site*

Site	Characteristics	Age (months)											
		9–12			13–16			17–20			21–24		
		Median		IQR	Median		IQR	Median		IQR	Median		IQR
BGD (*N* 234)	Weight (kg)												
	Mean		7·9			8·5			9·0			9·6	
	sd		1·0			1·1			1·1			1·2	
	Breastfed (% days)	99·2		99·2–99·2	99·2		99·2–99·2	99·2		99·2–99·2	99·2		99·1–99·2
	Energy intake from CF (kcal)	181		127–256	257		199–357	381		276–471	475		381–610
	Energy intake from breast milk (% total intake)	70		58–78	66		52–74	52		39–64	39		24–53
BRF (*N* 198)	Weight (kg)												
	Mean		10·0			10·9			11·7			12·3	
	sd		1·6			1·8			1·8			1·9	
	Breastfed (% days)	99·2		54·9–99·2	99·2		0·0–99·2	99·1		0·0–99·2	40·4		0·0–99·2
	Energy intake from CF (kcal)	822		629–1035	902		726–1092	1042		842–1226	1132		987–1255
	Energy intake from breast milk (% total intake)	9		0–24	6		0–23	3		0–14	0		0–10
INV (*N* 232)	Weight (kg)												
	Mean		7·5			8·2			8·9			9·5	
	sd		0·8			0·9			0·9			1·0	
	Breastfed (% days)	99·2		99·2–99·2	99·2		16·4–99·2	45·5		0·0–99·2	0·0		0·0–76·4
	Energy intake from CF (kcal)	461		331–613	629		472–835	818		664–1015	952		794–1149
	Energy intake from breast milk (% total intake)	25		6–43	18		0–37	1		0–17	0		0–1
PEL (*N* 251)	Weight (kg)												
	Mean		8·7			9·3			9·9			10·6	
	sd		1·1			1·1			1·1			1·1	
	Breastfed (% days)	99·2		99·2–99·2	99·2		87·3–99·2	61·1		0·0–99·2	0·0		0·0–51·6
	Energy intake from CF (kcal)	400		299–518	588		477–729	819		686–1026	982		808–1189
	Energy intake from breast milk (% total intake)	41		28–57	28		12–40	6		0–19	0		0–1
PKN (*N* 262)	Weight (kg)												
	Mean		7·7			8·3			8·9			9·6	
	sd		1·0			1·1			1·2			1·1	
	Breastfed (% days)	99·2		99·2–99·2	99·2		48·6–99·2	69·9		0·0–99·2	0·0		0·0–89·3
	Energy intake from CF (kcal)	350		240–532	506		365–699	704		500–919	847		669–1077
	Energy intake from breast milk (% total intake)	40		16–61	32		6–50	9		0–35	0		0–13
SAV (*N* 254)	Weight (kg)												
	Mean		8·6			9·4			10·2			10·9	
	sd		1·2			1·2			1·2			1·2	
	Breastfed (% days)	99·2		99·2–99·2	99·2		76·2–99·2	69·7		0·0–99·2	0·0		0·0–48·8
	Energy intake from CF (kcal)	562		408–765	752		569–941	940		753–1098	1108		917–1251
	Energy intake from breast milk (% total intake)	21		2–42	14		3–29	2		0–14	0		0–0
TZH (*N* 238)	Weight (kg)												
	Mean		8·2			8·8			9·4			10·0	
	sd		1·0			1·0			1·1			1·1	
	Breastfed (% days)	99·2		99·2–99·2	99·2		73·7–99·2	46·3		0·0–99·2	0·0		0·0–60·9
	Energy intake from CF (kcal)	761		623–956	911		752–1056	1036		877–1197	1174		1022–1334
	Energy intake from breast milk (% total intake)	4		0–14	4		0–11	0		0–6	0		0–0

BGD, Dhaka, Bangladesh; BRF, Fortaleza, Brazil; CF, complementary foods; INV, Vellore, India; PEL, Loreto, Peru; PKN, Naushero Feroze, Pakistan; SAV, Venda, South Africa; TZH, Haydom, Tanzania.

* To convert energy values from kilocalories to kilojoules, multiply it by 4·184.

**Fig. 1:**
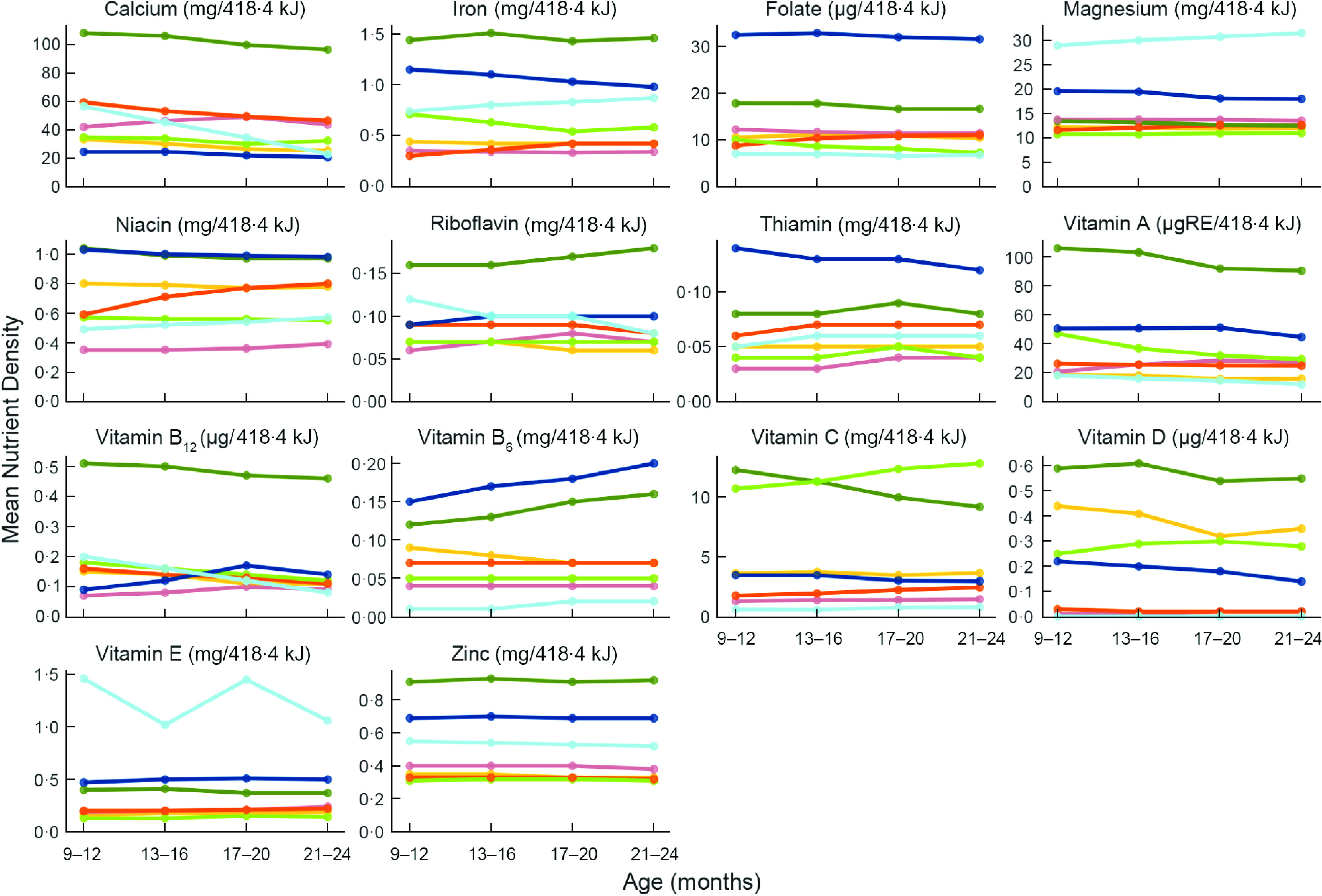
Mean nutrient densities (per 4·184 kJ or 100 kcal) of non-breast milk foods by age and site. BGD, Dhaka, Bangladesh; BRF, Fortaleza, Brazil; INV, Vellore, India; PEL, Loreto, Peru; PKN, Naushero Feroze, Pakistan; SAV, Venda, South Africa; TZH, Haydom, Tanzania. Site: 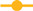, BGD; 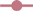, INV; 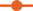, PKN; 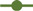, BRF; 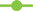, PEL; 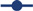, SAV; 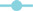, TZH

The mean nutrient densities from non-breast milk foods by site and age period are shown for each of the fourteen nutrients in Fig. 1 (with the values also included in Supplemental Table 2). Two general trends are worth noting. First, the mean nutrient densities in BRF and SAV were generally higher than in other sites, except for Mg density of the diet, which was highest in TZH. Second, the nutrient densities of the diet changed little as children aged from 9 to 24 months. Exceptions are the vitamin B_6_ density of the diet in SAV, which was going up; the decline in vitamin A and C densities in BRF; and the mean Ca, riboflavin and vitamin B_12_ densities in TZH, which were declining (see Supplemental Table 2 for mean values per nutrient by site and age period).

There were large differences across sites in the percentages of children consuming diets with a low bioavailability of zinc (Table 3). Only two children in BRF from 9 to 12 months, but 95–100 % of children in TZH at all age periods, were consuming a diet with low bioavailability. The percentages remained fairly constant across age periods, except for PEL and INV, where the data indicated that the bioavailability of zinc in the diet was increasing over the age range. As noted earlier, all children were deemed to have a diet with low bioavailability of iron based on the phytate-to-iron ratio.

**Table 3:** Percentage of child-days with low bioavailability* of zinc by site and age period

Site	Age (months)							
	*n*	%	*n*	%	*n*	%	*n*	%
	9–12		13–16		17–20		21–24	
South Asia								
BGD	72	7·3	65	6·7	90	9·8	88	9·8
INV	492	49·9	358	37·4	329	33·6	399	39·1
PKN	64	5·9	64	5·9	59	5·5	88	8·3
Latin America								
BRF	8	1·0	0	0·0	0	0·0	0	0·0
PEL	223	21·8	114	12·0	66	7·5	61	7·5
Sub-Saharan Africa								
SAV	837	84·5	847	83·3	851	84·1	811	80·7
TZH	913	95·5	950	97·8	962	100·0	889	100·0

BGD, Dhaka, Bangladesh; BRF, Fortaleza, Brazil; INV, Vellore, India; PEL, Loreto, Peru; PKN, Naushero Feroze, Pakistan; SAV, Venda, South Africa; TZH, Haydom, Tanzania.

* Low bioavailability was identified based on phytate-to-zinc ratio >15. Ref. (23).

**Fig. 2:**
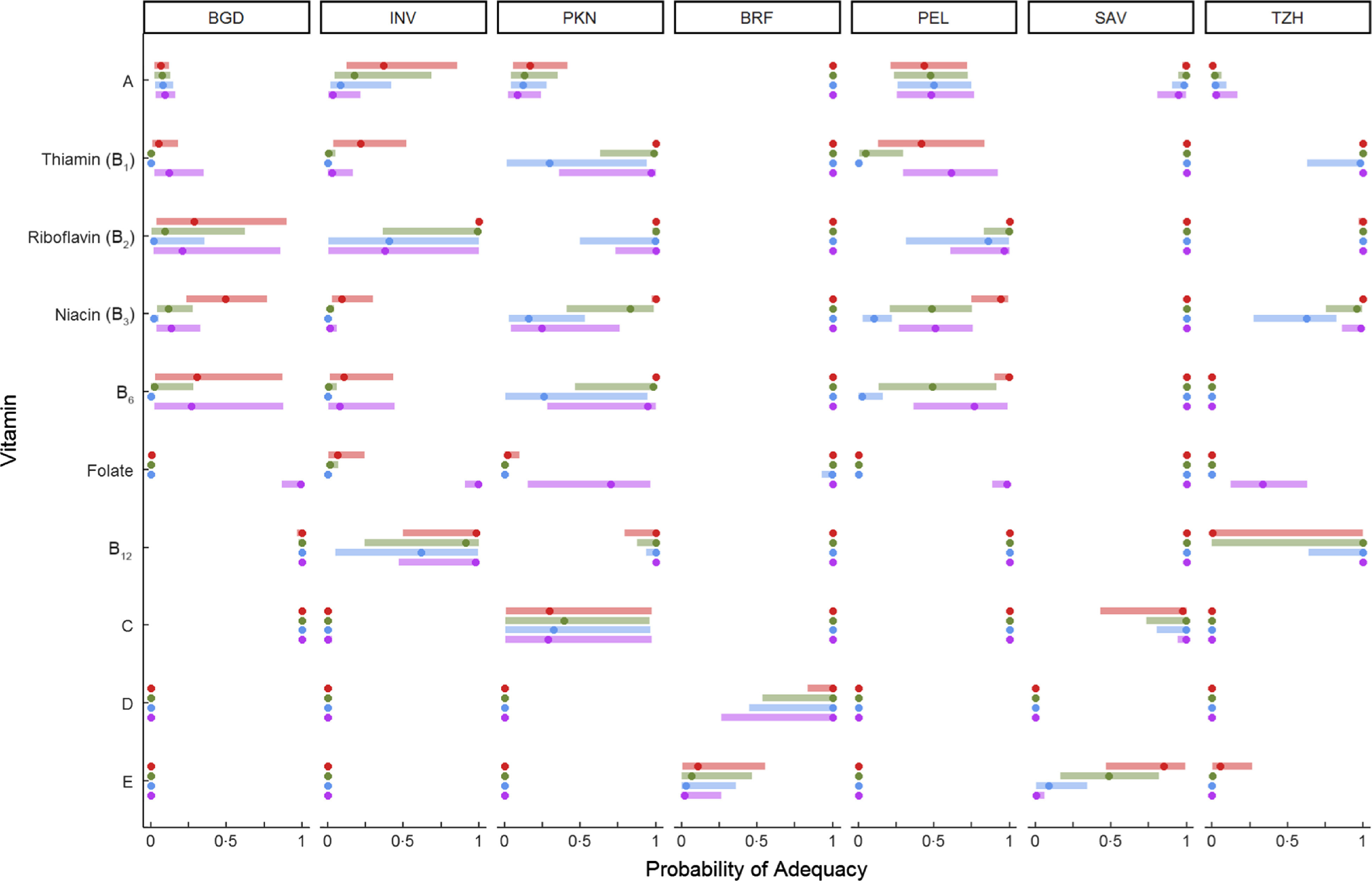
Median and IQR of the probability of adequacy of dietary vitamin intakes by age and site. BGD, Dhaka, Bangladesh; BRF, Fortaleza, Brazil; INV, Vellore, India; PEL, Loreto, Peru; PKN, Naushero Feroze, Pakistan; SAV, Venda, South Africa; TZH, Haydom, Tanzania. Age (months): 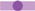, 9–12; 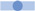, 13–16; 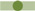, 17–20; 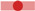, 21–24

**Fig. 3:**
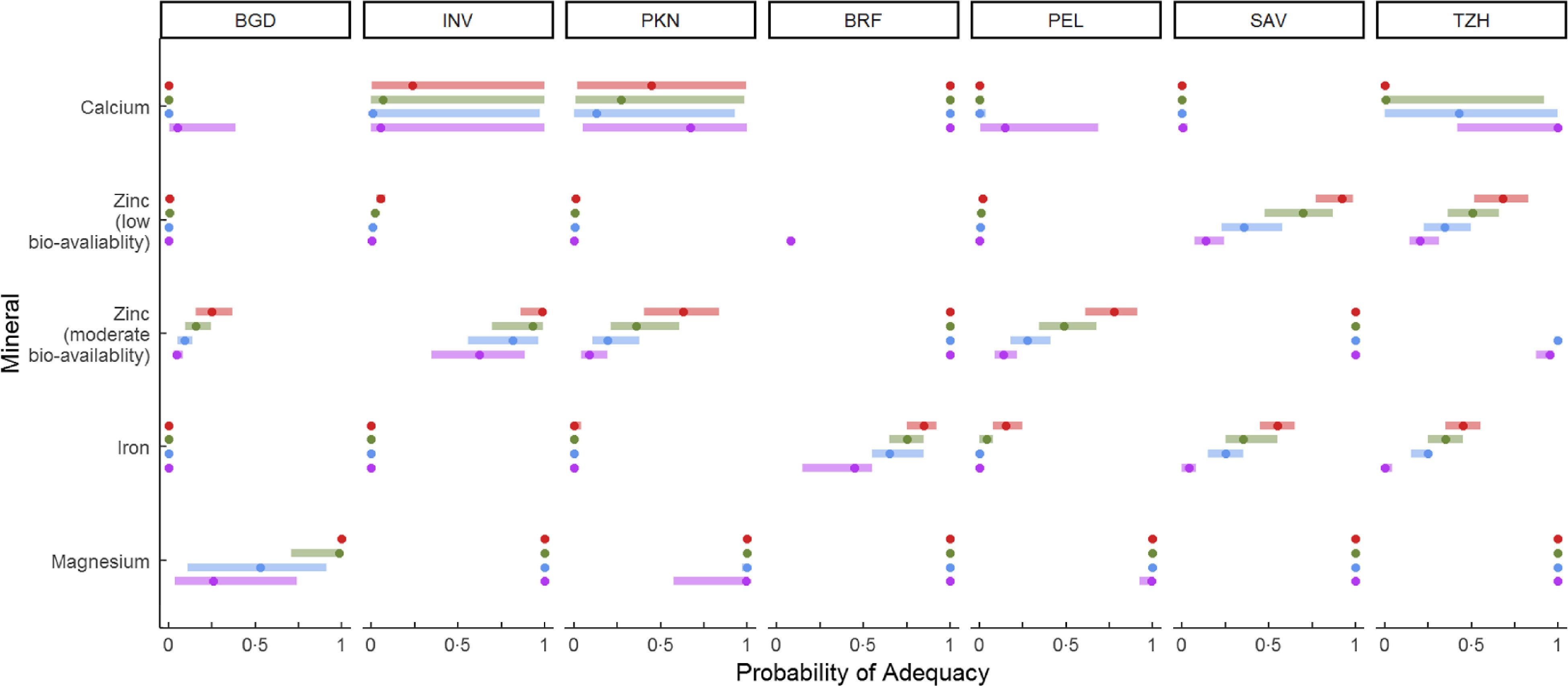
Median and IQR of the probability of adequacy of dietary mineral intakes by age and site. BGD, Dhaka, Bangladesh; BRF, Fortaleza, Brazil; INV, Vellore, India; PEL, Loreto, Peru; PKN, Naushero Feroze, Pakistan; SAV, Venda, South Africa; TZH, Haydom, Tanzania. Age (months): 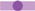, 9–12; 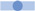, 13–16; 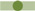, 17–20; 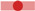, 21–24

The median (IQR) values of PA of each nutrient (from both breast milk and non-breast milk foods) by site and age period are shown in Figs. 2 (vitamins) and 3 (minerals) (see values by site in Supplemental Table 3). If PA were to be represented by only a filled circle (at either 0 or 1), this would mean that all children had the same value for PA at that age. The PA of zinc is shown in Fig. 3 separately by bioavailability.

### Trends in probability of adequacy by age

As children aged from 12 to 24 months, the median PA was increasing for many nutrients (see, e.g., the shift in median and IQR for vitamin A in INV, PEL and PKN). Age-associated trends of increasing PA were also seen for iron in some sites (BRF, SAV, TZH), and for zinc intakes in children consuming a diet with moderate bioavailability (INV, PEL, PKN), and for those in SAV and TZH consuming a diet with low bioavailability of zinc. In general, this is explained by the greater contribution of complementary foods to the total nutrient intake because, as noted above (and in Fig. 1), age-associated patterns of nutrient density of diet from non-breast milk foods were mostly flat.

It is also notable that the median PA of some nutrients, such as B vitamins, were higher before 12 months, lower for 13–16 months and then rose from there. The initial drop in PA was due to changes in EAR before and after 12 months (which can be up to a twofold increase in EAR at 12 months), whereas the rise in median PA from 13 to 24 months was related to age-related changes in the absolute amounts of nutrients consumed from non-breast milk foods in each site. The clearest example of this is folate, where it was true for six of seven sites. The shifts in EAR held across all sites; therefore, if the pattern was not observed (most B vitamins in BRF, riboflavin in PKN, B_6_ in INV), it may be because the reported amounts consumed from 12 to 24 months either did not reach the requirement distribution (folate in PEL) or they surpassed the requirement distribution irrespective of the age period (folate in BRF and SAV, median PA for riboflavin in PKN).

### Variability in probability of adequacy

For some nutrients, there was no variability in PA across children, and generally this occurred with a PA of 0 or 1. Nearly all children in both BRF and SAV had diets that were likely adequate for most nutrients (PA of 1). Exceptions to this were the PA of iron, vitamin D and vitamin E (both sites), calcium (SAV) and zinc (among the majority of children in SAV who were consuming a low bioavailable diet). A PA of 0 identified nutrients that were generally lacking in the diets of children. This was true for vitamin D for all sites except BRF, where vitamin D-fortified products were reported to be consumed for this age group; as shown, the median PA was 1 and the PA of the lower quartile increased with age. For vitamin E, the median PA was 0 as well, except for BRF and SAV, and TZH (at 21–24 months). A strong age-associated shift in PA for vitamin E in SAV was related to an increased consumption of foods sautéed in sunflower oil.

Overall, the median PA for vitamin B_12_ was 1 across sites, with the exception of INV and TZH. In these sites, PA was 1 at 9–12 months, but was lower thereafter and showed a large variability across children as evidenced by the IQR.

The width of the line reflects the width of the IQR of PA distribution for that nutrient. For vitamin A in BGD and PEL, it appeared constant across ages, but for many nutrients, the IQR broadened with increasing age (e.g. vitamin A in PKN). A wide variability could exist; examples included vitamin C in PKN, calcium in INV, PKN and TZH, riboflavin in BGD and INV, and B_6_ in BGD, PKN and PEL. This occured because of variability in the consumption of nutrient sources across children.

The age-associated trends and variability in PA for calcium in TZH were unusual and worth noting. Cow’s milk (e.g. fresh, soured, fermented) was an early and consistent source of calcium in the diets of these children, but as they aged from 12 to 24 months, it was less frequently fed and so consumption was more variable across children. Consistent with this, calcium, riboflavin and vitamin B_12_ nutrient densities declined from 12 to 24 months (Fig. 1), in part because the proportion of children no longer receiving milk increased. By 21–24 months, the median PA was 0 for calcium with little variability across children; the median PA for vitamin B_12_ was low but the upper quartile spanned to a PA of 1, reflecting a variability in the consumption of other sources of B_12_ among children.

### Mean probability of adequacy across fourteen nutrients

The distributions of MPA by site and age period are shown in Table 4. Differences in the patterns of PA by age and site, shown in Figs 2 and 3, led to the patterns of MPA. The median MPA increased as children aged, and two sites, BRF and SAV, had higher MPA than other sites.

**Table 4:** Mean probability of adequacy (MPA) of fourteen nutrients in the total intakes of children by age and site*

Site	Age (months)							
	9–12		13–16		17–20		21–24	
	Median	IQR	Median	IQR	Median	IQR	Median	IQR
South Asia								
BGD	0·34	0·25–0·43	0·20	0·16–0·26	0·26	0·20–0·32	0·34	0·26–0·41
INV	0·30	0·22–0·42	0·19	0·11–0·36	0·29	0·17–0·41	0·38	0·27–0·45
PKN	0·46	0·33–0·57	0·36	0·25–0·47	0·48	0·38–0·56	0·54	0·46–0·61
Latin America								
BRF	0·87	0·83–0·91	0·90	0·85–0·94	0·92	0·86–0·95	0·92	0·88–0·96
PEL	0·54	0·42–0·62	0·35	0·28–0·40	0·45	0·35–0·51	0·55	0·47–0·60
Sub-Saharan Africa								
SAV	0·65	0·63–0·70	0·69	0·66–0·74	0·74	0·70–0·79	0·78	0·73–0·82
TZH	0·46	0·41–0·49	0·37	0·31–0·44	0·39	0·34–0·46	0·40	0·36–0·45

BGD, Dhaka, Bangladesh; BRF, Fortaleza, Brazil; INV, Vellore, India; PEL, Loreto, Peru; PKN, Naushero Feroze, Pakistan; SAV, Venda, South Africa; TZH, Haydom, Tanzania.

* Presented are median and IQR of the distribution of child’s MPA.

### Sensitivity analysis

Although changes in the magnitude of MPA were found when considering non-breast milk foods only, especially in the first of the two age periods (9–12 and 13–16 months), the overall differences between sites were similar (online Supplemental Table 4). The MPA of the BGD site was much lower, consistent with the high proportion of breastfed children throughout the period and the estimated volumes of breast milk consumed (Table 2), as well as from the nutrients contributing to total intake and MPA in Table 4.

## Discussion

These analyses provide a unique characterisation of the adequacy of children’s diets in seven low- and medium-income country settings from South Asia, Sub-Saharan Africa and Latin America. Using a harmonised protocol, with 16–17 recalls per child, we quantified the intakes of non-breast milk foods, estimated the contribution of energy and micronutrients from breast milk, and utilised approaches to derive the estimates of usual nutrient intakes of children from 9 to 24 months of age. Our findings demonstrate the following four important patterns. First, with the exception of BRF and SAV, the adequacy of diets was generally low, even accounting for nutrients estimated to come from breast milk. Second, in general, the nutrient density of non-breast milk foods did not increase as children aged from 9 to 24 months. Third, the mean nutrient adequacy in each setting increased as children aged, largely because the overall amounts of complementary foods were increasing. Fourth, low bioavailability in some sites at some ages was the primary cause of low nutrient adequacy for zinc.

In SAV and BRF, the mean probability of nutrient adequacy ranged from 0·65–0·78 and 0·87–0·92, respectively. The nutrient PA for virtually all children in each of these sites was 1 for vitamins A, B_6_ and B_12_, thiamine, niacin, riboflavin, folate and zinc (if the diet was of medium bioavailability in SAV). These results can be largely explained by the presence of fortified food products in the diets of these children. Since 2003, the maize meal in South Africa, which is consumed almost daily by SAV children, has been fortified with vitamins A, B_6_, niacin, riboflavin, folate, zinc and iron^(27)^. Both white and brown bread flour are also fortified with these nutrients, but they are less frequently part of the diets of children in SAV. Commercial iron-fortified infant cereals are also available and reported to be consumed by children in SAV. Cow’s milk as well as multiple commercial cereal and milk products (e.g. follow-on formulas) for infants and children are available and widely consumed in BRF, and contributed to an overall high mean nutrient adequacy. Commercial milk and cereal products for infants and children were reported in the recalls at all of the MAL-ED sites, but apart from BRF and SAV, they were not frequently consumed.

Overall, the mean probability of nutrient adequacies in the other five sites were substantially lower across the three sites in South Asia (range 0·19–0·54), PEL (0·35–0·55) and TZH (0·37–0·46). Nutrient intakes, however, were not uniformly low. For some nutrients, nearly all children at all ages had a PA of 1: vitamin B_12_ (all sites but INV and TZH), vitamin C (all sites but INV and PKN), magnesium (all sites but BGD). For the remaining B vitamins, there was a great variability in nutrient adequacy across children, reflecting the variability in dietary sources consumed by children at each age period, but the PA for folate was 0 or near 0 in the second year of life in these sites. The adequacy of vitamin A was near 0 for most children in BGD and TZH regardless of age, whereas for the other sites, the adequacy improved with age. It is somewhat surprising that the adequacy for vitamin A in PEL was not closer to 1 given the tropical nature of the site and availability of fruits containing pro-vitamin A carotenoids. In terms of minerals, for iron, the PA was 0 for nearly all children at all ages, except for PEL and TZH. This was true for zinc as well as for children consuming a diet with low bioavailability. The PA was near 0 for calcium for BGD and PEL, but was highly variable for INV, PKN and TZH.

The data in Fig. 3 underscore the role of bioavailability in determining the PA for zinc. As shown, among children eating a diet with moderate bioavailability, the PA was increasing over time as intake from complementary foods increased, whereas for those consuming a diet with low bioavailability, increasing the amounts of complementary food did not increase the PA (except in SAV and TZH where maize meal was consumed daily). In BGD and PEL, increasing the zinc density of the diet would also be needed.

The nutrient density of the diet would likely increase during the second year of life due to greater fine motor skills, teeth eruption, expansion of appropriate foods items and as children consume family foods. Surprisingly, the mean nutrient density did not increase with age for most nutrients at most sites. Further analyses of these data to identify the nutrient sources and frequency of consumption leading to the greatest nutrient densities could lead to food-based recommendations to improve the adequacy for each nutrient across the age ranges in each site.

MAL-ED was a large study including about 200 children per site followed intensely over 24 months. Few studies have utilised repeated 24-h recalls to capture the non-breast milk dietary intakes of children, or had 4–5 recalls per child during 3-month age periods to quantify the adequacy of their diets and their progression over the majority of the period of complementary feeding. Our use of recommended statistical methods to estimate EUI and PA is another strength of the study. Limitations of the study can be found in the level of precision of nutrient contents of foods consumed across sites, but this is a limitation common to all dietary studies. The study could not quantify breast milk intakes and relied on published methods to estimate the energy and nutrient contribution of breast milk to the diets of these children. As well, our estimation of PA was based on published consensus on the nature and variability of nutrient requirements for young children, as well as procedures for characterising the mineral bioavailability of the diet.

Despite these limitations, our study provides novel insights into the adequacy of fourteen micronutrients in the total diets of children across these seven sites. We showed that the adequacy of the diet increased from 12 to 24 months as children consumed higher quantities of food. Ways to increase the consumption of vitamins D, E and A, and calcium are needed, as are ways to increase the bioavailability of iron and zinc. Further research is needed in these communities to understand the barriers to improved adequacy.

## Supplementary material

For supplementary material accompanying this paper visit https://doi.org/10.1017/S1368980020000877. click here to view supplementary material
