# Supplementary material for: Micronutrient intake and the probability of nutrient adequacy among children 9–24 months of age: results from the MAL-ED birth cohort study
Source: Public Health Nutr. 2020 Jul 2;24(9):2592–602. doi: 10.1017/S1368980020000877 (PMC8145466; doi:10.1017/S1368980020000877)
Supplement: Supplementary file 1 [file S1368980020000877sup001.docx]

Supplemental Table 1. Recommended Nutrient Intakes (RNIs), Coefficient of Variation (CV) and Estimated Average Requirement (EAR)

|  | **7-12 months** | | | **13-24** | | |
| --- | --- | --- | --- | --- | --- | --- |
|  | **RNI** | **CV** | **EAR** | **RNI** | **CV** | **EAR** |
| Thiamin | 0.30 | 0.10 | 0.25 | 0.50 | 0.10 | 0.42 |
| Riboflavin | 0.40 | 0.10 | 0.33 | 0.20 | 0.10 | 0.42 |
| Niacin | 4.00 | 0.15 | 3.08 | 6.00 | 0.15 | 4.62 |
| Vitamin B6 | 0.30 | 0.10 | 0.25 | 0.50 | 0.10 | 0.42 |
| Folate | 0.80 | 0.10 | 66.67 | 160.00 | 0.10 | 133.33 |
| Vitamin B12 | 0.70 | 0.10 | 0.58 | 0.90 | 0.10 | 0.75 |
| Vitamin A | 400.00 | 0.20 | 285.71 | 400.00 | 0.20 | 285.71 |
| Vitamin C | 30.00 | 0.10 | 25.00 | 30.00 | 0.10 | 25.00 |
| Vitamin D | 5.00 | 0.10 | 4.17 | 5.00 | 0.10 | 4.17 |
| Vitamin E* | 5.00 | - | - | 6.00 | 0.10 | 5.00 |
| Calcium | 400.00 | 0.10 | 333.33 | 500.00 | 0.10 | 416.67 |
| Iron (Low Bioavailability) ^ϯ^ | 18.60 | - | - | 11.60 | - | - |
| Zinc (Moderate Bioavailability) | 4.10 | 0.25 | 2.73 | 4.10 | 0.25 | 2.73 |
| Zinc (Low Bioavailability) | 8.40 | 0.25 | 5.60 | 8.30 | 0.25 | 5.53 |
| Magnesium | 54.00 | 0.10 | 45.00 | 60.00 | 0.10 | 50.00 |

RNIs taken from FAO/WHO (2004), except for Vitamin E (IOM, 2000)

CVs obtained from IOM (2001)

*RNI for Vitamin E in children 7-12 months not available, AI used instead.

^ϯ^ CV and EAR not available for Iron given its skewed distribution (IOM, 2001).

Supplemental Table 2. Mean nutrient densities per 100 kcal of non-breast milk foods by age and site

|  |  | Age (mo) | | | |
| --- | --- | --- | --- | --- | --- |
|  |  | 9-12 | 13-16 | 17-20 | 21-24 |
| Thiamin | BGD | 0.053 | 0.055 | 0.054 | 0.054 |
|  | BRF | 0.080 | 0.081 | 0.085 | 0.083 |
|  | INV | 0.032 | 0.033 | 0.041 | 0.039 |
|  | PEL | 0.042 | 0.042 | 0.048 | 0.043 |
|  | PKN | 0.060 | 0.069 | 0.074 | 0.074 |
|  | SAV | 0.139 | 0.133 | 0.125 | 0.124 |
|  | TZH | 0.051 | 0.056 | 0.055 | 0.058 |
| Riboflavin |  |  |  |  |  |
|  | BGD | 0.072 | 0.070 | 0.063 | 0.060 |
|  | BRF | 0.155 | 0.160 | 0.171 | 0.181 |
|  | INV | 0.058 | 0.068 | 0.075 | 0.069 |
|  | PEL | 0.070 | 0.068 | 0.068 | 0.067 |
|  | PKN | 0.094 | 0.092 | 0.089 | 0.085 |
|  | SAV | 0.088 | 0.096 | 0.100 | 0.095 |
|  | TZH | 0.116 | 0.104 | 0.097 | 0.080 |
| Niacin |  |  |  |  |  |
|  | BGD | 0.797 | 0.795 | 0.775 | 0.784 |
|  | BRF | 1.037 | 0.991 | 0.969 | 0.968 |
|  | INV | 0.345 | 0.346 | 0.364 | 0.394 |
|  | PEL | 0.574 | 0.557 | 0.557 | 0.549 |
|  | PKN | 0.591 | 0.707 | 0.775 | 0.798 |
|  | SAV | 1.032 | 1.001 | 0.987 | 0.984 |
|  | TZH | 0.494 | 0.522 | 0.544 | 0.574 |
|  |  |  |  |  |  |
| Vitamin B6 |  |  |  |  |  |
|  | BGD | 0.085 | 0.082 | 0.074 | 0.071 |
|  | BRF | 0.115 | 0.125 | 0.149 | 0.160 |
|  | INV | 0.037 | 0.039 | 0.038 | 0.038 |
|  | PEL | 0.054 | 0.053 | 0.052 | 0.051 |
|  | PKN | 0.067 | 0.070 | 0.074 | 0.074 |
|  | SAV | 0.153 | 0.173 | 0.184 | 0.200 |
|  | TZH | 0.012 | 0.013 | 0.017 | 0.020 |
| Folate |  |  |  |  |  |
|  | BGD | 10.437 | 11.042 | 10.788 | 10.360 |
|  | BRF | 17.751 | 17.716 | 16.554 | 16.555 |
|  | INV | 12.115 | 11.638 | 11.334 | 11.378 |
|  | PEL | 9.937 | 8.528 | 8.052 | 7.202 |
|  | PKN | 8.682 | 10.277 | 10.794 | 10.968 |
|  | SAV | 32.391 | 32.788 | 31.928 | 31.494 |
|  | TZH | 6.989 | 6.901 | 6.530 | 6.695 |
| Vitamin B12 |  |  |  |  |  |
|  | BGD | 0.151 | 0.140 | 0.114 | 0.110 |
|  | BRF | 0.507 | 0.499 | 0.472 | 0.462 |
|  | INV | 0.068 | 0.082 | 0.101 | 0.092 |
|  | PEL | 0.182 | 0.156 | 0.144 | 0.124 |
|  | PKN | 0.163 | 0.144 | 0.126 | 0.110 |
|  | SAV | 0.093 | 0.123 | 0.172 | 0.139 |
|  | TZH | 0.198 | 0.158 | 0.119 | 0.075 |
| Vitamin A |  |  |  |  |  |
|  | BGD | 18.490 | 17.928 | 15.408 | 15.611 |
|  | BRF | 105.836 | 103.049 | 91.770 | 90.272 |
|  | INV | 20.568 | 25.456 | 28.365 | 26.879 |
|  | PEL | 46.781 | 36.707 | 31.794 | 29.199 |
|  | PKN | 26.076 | 25.451 | 24.750 | 24.613 |
|  | SAV | 50.363 | 50.491 | 50.959 | 44.447 |
|  | TZH | 17.948 | 15.812 | 14.400 | 11.793 |
| Vitamin C |  |  |  |  |  |
|  | BGD | 3.629 | 3.745 | 3.485 | 3.656 |
|  | BRF | 12.315 | 11.352 | 9.988 | 9.209 |
|  | INV | 1.301 | 1.376 | 1.393 | 1.455 |
|  | PEL | 10.751 | 11.326 | 12.395 | 12.864 |
|  | PKN | 1.772 | 1.952 | 2.242 | 2.457 |
|  | SAV | 3.481 | 3.470 | 3.030 | 2.984 |
|  | TZH | 0.612 | 0.578 | 0.765 | 0.790 |
|  |  |  |  |  |  |
| Vitamin D | BGD | 0.436 | 0.415 | 0.322 | 0.353 |
|  | BRF | 0.585 | 0.613 | 0.541 | 0.550 |
|  | INV | 0.011 | 0.014 | 0.022 | 0.022 |
|  | PEL | 0.250 | 0.286 | 0.299 | 0.281 |
|  | PKN | 0.025 | 0.022 | 0.023 | 0.020 |
|  | SAV | 0.218 | 0.196 | 0.180 | 0.138 |
|  | TZH | 0.004 | 0.004 | 0.004 | 0.003 |
| Vitamin E |  |  |  |  |  |
|  | BGD | 0.159 | 0.178 | 0.174 | 0.190 |
|  | BRF | 0.404 | 0.411 | 0.373 | 0.374 |
|  | INV | 0.187 | 0.198 | 0.214 | 0.244 |
|  | PEL | 0.130 | 0.130 | 0.146 | 0.139 |
|  | PKN | 0.201 | 0.199 | 0.205 | 0.220 |
|  | SAV | 0.467 | 0.498 | 0.515 | 0.500 |
|  | TZH | 1.458 | 1.017 | 1.447 | 1.065 |
|  |  |  |  |  |  |
| Calcium | BGD | 33.082 | 29.980 | 26.298 | 25.109 |
|  | BRF | 108.115 | 106.081 | 99.737 | 96.475 |
|  | INV | 41.839 | 46.135 | 48.969 | 43.522 |
|  | PEL | 34.491 | 33.666 | 29.819 | 32.164 |
|  | PKN | 59.215 | 53.055 | 49.301 | 46.401 |
|  | SAV | 24.314 | 24.363 | 21.759 | 20.446 |
|  | TZH | 56.341 | 45.011 | 34.310 | 22.790 |
|  |  |  |  |  |  |
| Iron | BGD | 0.438 | 0.422 | 0.417 | 0.423 |
|  | BRF | 1.440 | 1.507 | 1.425 | 1.463 |
|  | INV | 0.347 | 0.339 | 0.333 | 0.340 |
|  | PEL | 0.710 | 0.628 | 0.537 | 0.584 |
|  | PKN | 0.301 | 0.365 | 0.416 | 0.418 |
|  | SAV | 1.155 | 1.103 | 1.033 | 0.985 |
|  | TZH | 0.738 | 0.797 | 0.833 | 0.874 |
| Zinc |  |  |  |  |  |
|  | BGD | 0.353 | 0.350 | 0.331 | 0.329 |
|  | BRF | 0.909 | 0.933 | 0.914 | 0.922 |
|  | INV | 0.397 | 0.402 | 0.401 | 0.383 |
|  | PEL | 0.313 | 0.316 | 0.324 | 0.313 |
|  | PKN | 0.328 | 0.330 | 0.328 | 0.323 |
|  | SAV | 0.688 | 0.701 | 0.690 | 0.688 |
|  | TZH | 0.550 | 0.541 | 0.525 | 0.515 |
| Magnesium |  |  |  |  |  |
|  | BGD | 12.202 | 12.031 | 11.849 | 11.926 |
|  | BRF | 13.425 | 13.103 | 12.626 | 12.454 |
|  | INV | 13.714 | 13.735 | 13.668 | 13.511 |
|  | PEL | 10.681 | 10.664 | 10.898 | 10.936 |
|  | PKN | 11.516 | 12.074 | 12.569 | 12.571 |
|  | SAV | 19.540 | 19.435 | 18.056 | 17.943 |
|  | TZH | 28.970 | 30.018 | 30.749 | 31.513 |

Abbreviations used: BGD, Bangladesh; BRF, Brazil; INV, India; PEL, Peru; PKN, Pakistan; SAV, South Africa; TZH, Tanzania.

Supplemental Table 3. Nutrient Probability of Adequacy (median and interquartile range) by Age group and Site.

|  | **BGD** | | | |
| --- | --- | --- | --- | --- |
|  | **9-12** | **13-16** | **17-20** | **21-24** |
| Thiamin | 0.12 (0.02-0.35) | 0.00 (0.00-0.00) | 0.00 (0.00-0.02) | 0.05 (0.01-0.18) |
| Riboflavin | 0.21 (0.02-0.86) | 0.02 (0.00-0.36) | 0.09 (0.01-0.63) | 0.29 (0.04-0.90) |
| Niacin | 0.14 (0.04-0.33) | 0.02 (0.00-0.05) | 0.12 (0.04-0.28) | 0.49 (0.24-0.77) |
| Pyridoxine | 0.27 (0.02-0.87) | 0.00 (0.00-0.02) | 0.03 (0.00-0.28) | 0.30 (0.03-0.87) |
| Folate | 0.99 (0.87-1.00) | 0.00 (0.00-0.00) | 0.00 (0.00-0.01) | 0.00 (0.00-0.02) |
| Cobalamin | 1.00 (1.00-1.00) | 1.00 (0.99-1.00) | 1.00 (0.98-1.00) | 1.00 (0.97-1.00) |
| Vit. C | 1.00 (1.00-1.00) | 1.00 (1.00-1.00) | 1.00 (1.00-1.00) | 1.00 (1.00-1.00) |
| Vit. A | 0.09 (0.03-0.16) | 0.08 (0.03-0.15) | 0.07 (0.03-0.13) | 0.06 (0.02-0.12) |
| Calcium | 0.05 (0.00-0.39) | 0.00 (0.00-0.01) | 0.00 (0.00-0.01) | 0.00 (0.00-0.01) |
| Iron | 0.00 (0.00-0.00) | 0.00 (0.00-0.00) | 0.00 (0.00-0.00) | 0.00 (0.00-0.00) |
| Low Bio Zinc | 0.00 (0.00-0.00) | 0.00 (0.00-0.00) | 0.00 (0.00-0.00) | 0.01 (0.00-0.01) |
| Mod Bio Zinc | 0.05 (0.03-0.08) | 0.09 (0.05-0.14) | 0.16 (0.10-0.24) | 0.25 (0.16-0.37) |
| Magnesium | 0.26 (0.03-0.74) | 0.53 (0.11-0.91) | 0.98 (0.70-1.00) | 1.00 (1.00-1.00) |
| Vit. E | 0.00 (0.00-0.00) | 0.00 (0.00-0.00) | 0.00 (0.00-0.00) | 0.00 (0.00-0.00) |
| Vit. D | 0.00 (0.00-0.00) | 0.00 (0.00-0.00) | 0.00 (0.00-0.00) | 0.00 (0.00-0.00) |
|  | **BRF** | | | |
| Thiamin | 1.00 (1.00-1.00) | 1.00 (1.00-1.00) | 1.00 (1.00-1.00) | 1.00 (1.00-1.00) |
| Riboflavin | 1.00 (1.00-1.00) | 1.00 (1.00-1.00) | 1.00 (1.00-1.00) | 1.00 (1.00-1.00) |
| Niacin | 1.00 (1.00-1.00) | 1.00 (1.00-1.00) | 1.00 (1.00-1.00) | 1.00 (1.00-1.00) |
| Pyridoxine | 1.00 (1.00-1.00) | 1.00 (1.00-1.00) | 1.00 (1.00-1.00) | 1.00 (1.00-1.00) |
| Folate | 1.00 (1.00-1.00) | 1.00 (0.93-1.00) | 1.00 (0.99-1.00) | 1.00 (1.00-1.00) |
| Cobalamin | 1.00 (1.00-1.00) | 1.00 (1.00-1.00) | 1.00 (1.00-1.00) | 1.00 (1.00-1.00) |
| Vit. C | 1.00 (1.00-1.00) | 1.00 (1.00-1.00) | 1.00 (1.00-1.00) | 1.00 (1.00-1.00) |
| Vit. A | 1.00 (1.00-1.00) | 1.00 (1.00-1.00) | 1.00 (1.00-1.00) | 1.00 (1.00-1.00) |
| Calcium | 1.00 (1.00-1.00) | 1.00 (1.00-1.00) | 1.00 (1.00-1.00) | 1.00 (1.00-1.00) |
| Iron | 0.45 (0.15-0.55) | 0.65 (0.55-0.85) | 0.75 (0.65-0.85) | 0.85 (0.75-0.92) |
| Low Bio Zinc | 0.08 (0.04-0.11) | (-) | (-) | (-) |
| Mod Bio Zinc | 1.00 (1.00-1.00) | 1.00 (1.00-1.00) | 1.00 (1.00-1.00) | 1.00 (1.00-1.00) |
| Magnesium | 1.00 (1.00-1.00) | 1.00 (1.00-1.00) | 1.00 (1.00-1.00) | 1.00 (1.00-1.00) |
| Vit. E | 0.02 (0.00-0.27) | 0.03 (0.00-0.36) | 0.07 (0.00-0.47) | 0.11 (0.00-0.56) |
| Vit. D | 1.00 (0.27-1.00) | 1.00 (0.43-1.00) | 1.00 (0.52-1.00) | 1.00 (0.83-1.00) |
|  | **INV** | | | |
| Thiamin | 0.03 (0.00-0.17) | 0.00 (0.00-0.00) | 0.01 (0.00-0.05) | 0.22 (0.04-0.52) |
| Riboflavin | 0.38 (0.00-1.00) | 0.41 (0.01-1.00) | 0.99 (0.37-1.00) | 1.00 (0.98-1.00) |
| Niacin | 0.02 (0.01-0.06) | 0.00 (0.00-0.01) | 0.01 (0.00-0.04) | 0.10 (0.03-0.30) |
| Pyridoxine | 0.08 (0.01-0.45) | 0.00 (0.00-0.00) | 0.01 (0.00-0.06) | 0.11 (0.01-0.44) |
| Folate | 1.00 (0.91-1.00) | 0.00 (0.00-0.01) | 0.01 (0.00-0.07) | 0.07 (0.01-0.25) |
| Cobalamin | 0.98 (0.46-1.00) | 0.62 (0.05-1.00) | 0.91 (0.24-1.00) | 0.98 (0.50-1.00) |
| Vit. C | 0.00 (0.00-0.03) | 0.00 (0.00-0.01) | 0.00 (0.00-0.01) | 0.00 (0.00-0.00) |
| Vit. A | 0.03 (0.01-0.22) | 0.08 (0.02-0.42) | 0.17 (0.04-0.69) | 0.37 (0.12-0.86) |
| Calcium | 0.05 (0.00-1.00) | 0.01 (0.00-0.97) | 0.07 (0.00-1.00) | 0.24 (0.00-1.00) |
| Iron | 0.00 (0.00-0.00) | 0.00 (0.00-0.00) | 0.00 (0.00-0.00) | 0.00 (0.00-0.00) |
| Low Bio Zinc | 0.00 (0.00-0.01) | 0.01 (0.01-0.01) | 0.02 (0.01-0.03) | 0.05 (0.03-0.08) |
| Mod Bio Zinc | 0.62 (0.34-0.89) | 0.82 (0.56-0.96) | 0.93 (0.70-0.99) | 0.99 (0.86-1.00) |
| Magnesium | 1.00 (1.00-1.00) | 1.00 (1.00-1.00) | 1.00 (1.00-1.00) | 1.00 (1.00-1.00) |
| Vit. E | 0.00 (0.00-0.00) | 0.00 (0.00-0.00) | 0.00 (0.00-0.00) | 0.00 (0.00-0.00) |
| Vit. D | 0.00 (0.00-0.00) | 0.00 (0.00-0.00) | 0.00 (0.00-0.00) | 0.00 (0.00-0.00) |
|  | **PEL** | | | |
| Thiamin | 0.61 (0.30-0.92) | 0.00 (0.00-0.02) | 0.05 (0.01-0.30) | 0.42 (0.13-0.84) |
| Riboflavin | 0.97 (0.61-1.00) | 0.86 (0.31-1.00) | 1.00 (0.83-1.00) | 1.00 (1.00-1.00) |
| Niacin | 0.51 (0.27-0.76) | 0.10 (0.03-0.22) | 0.48 (0.21-0.76) | 0.94 (0.75-0.99) |
| Pyridoxine | 0.77 (0.36-0.99) | 0.02 (0.00-0.16) | 0.49 (0.13-0.92) | 1.00 (0.90-1.00) |
| Folate | 0.99 (0.88-1.00) | 0.00 (0.00-0.00) | 0.00 (0.00-0.00) | 0.00 (0.00-0.00) |
| Cobalamin | 1.00 (1.00-1.00) | 1.00 (1.00-1.00) | 1.00 (1.00-1.00) | 1.00 (1.00-1.00) |
| Vit. C | 1.00 (1.00-1.00) | 1.00 (1.00-1.00) | 1.00 (1.00-1.00) | 1.00 (1.00-1.00) |
| Vit. A | 0.48 (0.25-0.77) | 0.50 (0.26-0.75) | 0.48 (0.23-0.73) | 0.43 (0.21-0.72) |
| Calcium | 0.15 (0.00-0.70) | 0.00 (0.00-0.04) | 0.00 (0.00-0.02) | 0.00 (0.00-0.02) |
| Iron | 0.00 (0.00-0.00) | 0.00 (0.00-0.00) | 0.04 (0.00-0.08) | 0.15 (0.08-0.25) |
| Low Bio Zinc | 0.00 (0.00-0.00) | 0.01 (0.00-0.01) | 0.01 (0.01-0.01) | 0.02 (0.01-0.03) |
| Mod Bio Zinc | 0.14 (0.09-0.22) | 0.28 (0.18-0.41) | 0.49 (0.34-0.68) | 0.78 (0.61-0.91) |
| Magnesium | 1.00 (0.92-1.00) | 1.00 (0.99-1.00) | 1.00 (1.00-1.00) | 1.00 (1.00-1.00) |
| Vit. E | 0.00 (0.00-0.00) | 0.00 (0.00-0.00) | 0.00 (0.00-0.00) | 0.00 (0.00-0.00) |
| Vit. D | 0.00 (0.00-0.00) | 0.00 (0.00-0.00) | 0.00 (0.00-0.00) | 0.00 (0.00-0.00) |
|  | **PKN** | | | |
| Thiamin | 0.97 (0.34-1.00) | 0.29 (0.02-0.94) | 0.99 (0.63-1.00) | 1.00 (1.00-1.00) |
| Riboflavin | 1.00 (0.73-1.00) | 1.00 (0.49-1.00) | 1.00 (0.98-1.00) | 1.00 (1.00-1.00) |
| Niacin | 0.25 (0.04-0.76) | 0.16 (0.03-0.53) | 0.83 (0.41-0.99) | 1.00 (0.97-1.00) |
| Pyridoxine | 0.95 (0.28-1.00) | 0.26 (0.01-0.95) | 0.98 (0.47-1.00) | 1.00 (1.00-1.00) |
| Folate | 0.70 (0.15-0.97) | 0.00 (0.00-0.00) | 0.00 (0.00-0.01) | 0.02 (0.00-0.10) |
| Cobalamin | 1.00 (1.00-1.00) | 1.00 (0.93-1.00) | 1.00 (0.88-1.00) | 1.00 (0.79-1.00) |
| Vit. C | 0.29 (0.01-0.97) | 0.32 (0.01-0.96) | 0.39 (0.01-0.96) | 0.29 (0.01-0.98) |
| Vit. A | 0.09 (0.03-0.24) | 0.12 (0.04-0.28) | 0.13 (0.04-0.35) | 0.17 (0.06-0.42) |
| Calcium | 0.67 (0.05-1.00) | 0.13 (0.00-0.93) | 0.27 (0.01-0.99) | 0.45 (0.02-0.99) |
| Iron | 0.00 (0.00-0.00) | 0.00 (0.00-0.00) | 0.00 (0.00-0.00) | 0.00 (0.00-0.04) |
| Low Bio Zinc | 0.00 (0.00-0.00) | 0.00 (0.00-0.01) | 0.01 (0.00-0.01) | 0.01 (0.01-0.01) |
| Mod Bio Zinc | 0.09 (0.04-0.19) | 0.19 (0.11-0.38) | 0.36 (0.21-0.61) | 0.63 (0.40-0.84) |
| Magnesium | 0.99 (0.57-1.00) | 1.00 (0.97-1.00) | 1.00 (1.00-1.00) | 1.00 (1.00-1.00) |
| Vit. E | 0.00 (0.00-0.00) | 0.00 (0.00-0.00) | 0.00 (0.00-0.00) | 0.00 (0.00-0.00) |
| Vit. D | 0.00 (0.00-0.00) | 0.00 (0.00-0.00) | 0.00 (0.00-0.00) | 0.00 (0.00-0.00) |
|  | **SAV** | | | |
| Thiamin | 1.00 (1.00-1.00) | 1.00 (1.00-1.00) | 1.00 (1.00-1.00) | 1.00 (1.00-1.00) |
| Riboflavin | 1.00 (1.00-1.00) | 1.00 (1.00-1.00) | 1.00 (1.00-1.00) | 1.00 (1.00-1.00) |
| Niacin | 1.00 (1.00-1.00) | 1.00 (1.00-1.00) | 1.00 (1.00-1.00) | 1.00 (1.00-1.00) |
| Pyridoxine | 1.00 (1.00-1.00) | 1.00 (1.00-1.00) | 1.00 (1.00-1.00) | 1.00 (1.00-1.00) |
| Folate | 1.00 (1.00-1.00) | 1.00 (1.00-1.00) | 1.00 (1.00-1.00) | 1.00 (1.00-1.00) |
| Cobalamin | 1.00 (1.00-1.00) | 1.00 (1.00-1.00) | 1.00 (1.00-1.00) | 1.00 (1.00-1.00) |
| Vit. C | 1.00 (0.93-1.00) | 1.00 (0.80-1.00) | 1.00 (0.74-1.00) | 0.98 (0.43-1.00) |
| Vit. A | 0.95 (0.81-1.00) | 0.99 (0.91-1.00) | 1.00 (0.95-1.00) | 1.00 (0.97-1.00) |
| Calcium | 0.00 (0.00-0.03) | 0.00 (0.00-0.00) | 0.00 (0.00-0.00) | 0.00 (0.00-0.00) |
| Iron | 0.04 (0.00-0.08) | 0.25 (0.15-0.35) | 0.35 (0.25-0.55) | 0.55 (0.45-0.65) |
| Low Bio Zinc | 0.14 (0.07-0.24) | 0.36 (0.23-0.58) | 0.70 (0.48-0.87) | 0.92 (0.77-0.99) |
| Mod Bio Zinc | 1.00 (1.00-1.00) | 1.00 (1.00-1.00) | 1.00 (1.00-1.00) | 1.00 (1.00-1.00) |
| Magnesium | 1.00 (1.00-1.00) | 1.00 (1.00-1.00) | 1.00 (1.00-1.00) | 1.00 (1.00-1.00) |
| Vit. E | 0.01 (0.00-0.06) | 0.09 (0.01-0.35) | 0.49 (0.17-0.82) | 0.85 (0.47-0.99) |
| Vit. D | 0.00 (0.00-0.00) | 0.00 (0.00-0.00) | 0.00 (0.00-0.00) | 0.00 (0.00-0.00) |
|  | **TZH** | | | |
| Thiamin | 1.00 (1.00-1.00) | 0.98 (0.63-1.00) | 1.00 (0.99-1.00) | 1.00 (1.00-1.00) |
| Riboflavin | 1.00 (1.00-1.00) | 1.00 (1.00-1.00) | 1.00 (1.00-1.00) | 1.00 (0.97-1.00) |
| Niacin | 0.98 (0.86-1.00) | 0.63 (0.27-0.82) | 0.96 (0.75-0.99) | 1.00 (0.99-1.00) |
| Pyridoxine | 0.00 (0.00-0.00) | 0.00 (0.00-0.00) | 0.00 (0.00-0.00) | 0.00 (0.00-0.00) |
| Folate | 0.34 (0.12-0.63) | 0.00 (0.00-0.00) | 0.00 (0.00-0.00) | 0.00 (0.00-0.00) |
| Cobalamin | 1.00 (1.00-1.00) | 1.00 (0.60-1.00) | 1.00 (0.00-1.00) | 0.00 (0.00-1.00) |
| Vit. C | 0.00 (0.00-0.00) | 0.00 (0.00-0.00) | 0.00 (0.00-0.00) | 0.00 (0.00-0.00) |
| Vit. A | 0.02 (0.01-0.17) | 0.02 (0.00-0.10) | 0.02 (0.00-0.06) | 0.00 (0.00-0.02) |
| Calcium | 1.00 (0.41-1.00) | 0.43 (0.00-1.00) | 0.00 (0.00-0.93) | 0.00 (0.00-0.01) |
| Iron | 0.00 (0.00-0.04) | 0.25 (0.15-0.25) | 0.35 (0.25-0.45) | 0.45 (0.35-0.55) |
| Low Bio Zinc | 0.20 (0.14-0.31) | 0.34 (0.23-0.50) | 0.51 (0.36-0.66) | 0.68 (0.51-0.83) |
| Mod Bio Zinc | 0.95 (0.85-0.98) | 1.00 (0.98-1.00) | (-) | (-) |
| Magnesium | 1.00 (1.00-1.00) | 1.00 (1.00-1.00) | 1.00 (1.00-1.00) | 1.00 (1.00-1.00) |
| Vit. E | 0.00 (0.00-0.00) | 0.00 (0.00-0.00) | 0.00 (0.00-0.01) | 0.05 (0.00-0.27) |
| Vit. D | 0.00 (0.00-0.00) | 0.00 (0.00-0.00) | 0.00 (0.00-0.00) | 0.00 (0.00-0.00) |

Abbreviations used: BGD, Bangladesh; BRF, Brazil; INV, India; PEL, Peru; PKN, Pakistan; SAV, South Africa; TZH, Tanzania; Mod Bio, moderate bioavailability.

**Supplemental Table 4. Mean probability of adequacy (MPA) of 14 nutrients in the total intakes of children by age and site using non-breast milk foods only.**

|  | **Age (mo)** | | | |
| --- | --- | --- | --- | --- |
| **Site** | **9-12** | **13-16** | **17-20** | **21-24** |
| BGD | 0.00 (0.00-0.03) | 0.00 (0.00-0.02) | 0.02 (0.00-0.11) | 0.11 (0.04-0.25) |
| INV | 0.12 (0.07-0.30) | 0.11 (0.07-0.30) | 0.23 (0.10-0.41) | 0.38 (0.21-0.49) |
| PKN | 0.21 (0.01-0.38) | 0.22 (0.04-0.37) | 0.37 (0.22-0.53) | 0.50 (0.38-0.64) |
| BRF | 0.87 (0.82-0.90) | 0.89 (0.82-0.93) | 0.90 (0.84-0.94) | 0.91 (0.87-0.96) |
| PEL | 0.15 (0.06-0.29) | 0.21 (0.12-0.30) | 0.39 (0.29-0.50) | 0.62 (0.50-0.69) |
| SAV | 0.50 (0.45-0.59) | 0.58 (0.51-0.67) | 0.70 (0.61-0.79) | 0.79 (0.72-0.83) |
| TZH | 0.41 (0.34-0.46) | 0.35 (0.29-0.43) | 0.35 (0.29-0.43) | 0.42 (0.35-0.47) |

Abbreviations used: BGD, Bangladesh; BRF, Brazil; INV, India; PEL, Peru; PKN, Pakistan; SAV, South Africa; TZH, Tanzania. Presented are median (interquartile range) of the distribution of child MPA.
